# Lower Limb Arthroplasties in Colombia: Projections for 2050 Based on Official Records

**DOI:** 10.3390/epidemiologia6020024

**Published:** 2025-05-08

**Authors:** Yesika Natali Fernández-Ortiz, Jorge Martín Rodríguez-Hernández

**Affiliations:** Pontificia Universidad Javeriana, Institute of Public Health, Bogotá 110231, Colombia; jrodriguez.h@javeriana.edu.co

**Keywords:** aging, arthroplasty, health data, forecasting, Colombia

## Abstract

Population ageing is driving a growing demand for orthopedic surgical procedures. The rise in chronic conditions such as osteoarthritis significantly contributes to disability among older adults, particularly women, and primarily affects the hip and knee joints, thereby increasing the need for arthroplasties. Objective: To determine the future demand for lower limb arthroplasty procedures among individuals aged 60 and over in Colombia up to 2050, using official public health records and national demographic projections. Methods: This study used an observational longitudinal retrospective design, using a Poisson regression model with official records from the Integrated Social Protection Information System—which consolidates procedures reported by both public and private healthcare service providers—to identify lower limb arthroplasties performed between 2015 and 2023. Population projections from the National Department of Statistics were incorporated to model future demand, accounting for demographic ageing and mortality trends. An additional analysis was conducted by sex and the most prevalent types of arthroplasties. Results: A total of 62,728 procedures took place from 2015 to 2023, with women undergoing approximately twice as many as men. The highest intervention rates occurred in the 65–69 and ≥80 age groups. By 2050, projections indicate the number of procedures will reach 39,270, with 52.7% projected among women. Conclusions: This study reports demographic trends in arthroplasties between 2015 and 2023 and offers insights into the anticipated future burden of lower limb arthroplasties among Colombia’s older population.

## 1. Introduction

Ongoing demographic trends towards population ageing reflect a combination of increased life expectancy and declining fertility rates, resulting in a marked shift in the age structure of many societies [1]. Consequently, population ageing has become a global phenomenon, posing a variety of public health challenges. Chief among these is the rising prevalence of chronic and degenerative diseases, particularly those affecting the musculoskeletal system [2,3]. Within this group, osteoarthritis and other joint disorders are leading causes of disability and diminished quality of life in older adults [3].

According to the Global Burden of Disease, Injuries, and Risk Factors (GBD) report, in 2019, musculoskeletal disorders ranked as the second leading cause of non-fatal disability, resulting in 322.75 million incident cases, 117,540 deaths, and 150.08 million Disability-Adjusted Life Years (DALYs) in 2019 [4,5]. Osteoarthritis is a major contributor to years lived with disability, with prevalence rising sharply with age and peaking in the 60–64 age group [5]. The GBD further reported that 12.46% of incident cases resulted in 19 million Years Lived with Disability (YLDs) [3,5].

Previous studies predicted that approximately 595 million people worldwide would suffer from osteoarthritis, representing 7.6% of the global population and an increase of 132.2% since 1990 [6]. By 2050, estimates suggest that cases of osteoarthritis will rise by 74.9% in knees, 78.6% in hips, 48.6% in hands, and 95.1% in other forms of osteoarthritis, particularly among women [6].

To address osteoarthritis—a degenerative joint disease that causes pain, stiffness, and reduced functionality, significantly impacting the quality of life of those affected—conservative treatment is initially preferred, such as anti-inflammatory medications and physiotherapy. However, when these measures fail to provide sufficient relief, arthroplasties become a viable and effective option [7].

Arthroplasties involve the surgical replacement or repair of joints using prosthetic components, with the primary aim of alleviating pain and restoring joint function in patients with severe damage [8]. When complications such as wear, infection, or mechanical problems arise in an existing prosthesis, revision procedures address these issues [9,10]. These interventions are essential for preserving the long-term benefits of joint replacement surgeries, ensuring improved mobility and quality of life for affected individuals.

Among these procedures, hip and knee arthroplasties stand out as lasting solutions, proven to restore mobility and reduce pain, enabling patients to resume daily activities with fewer limitations [11,12]. However, the increasing demand for these procedures poses significant challenges for healthcare resource planning and management.

Several studies in high-income countries projected future demand for hip and knee arthroplasties using official health records and modelling techniques. For instance, research from the United States, the United Kingdom, and Scotland has applied Poisson or time-series models to estimate the increasing volume of procedures expected in ageing populations [13,14,15,16,17,18,19,20]. Such projections have guided healthcare planning and the estimation of surgical workforce needs. However, comparable projection studies remain notably scarce in Latin America, particularly those based on nationally representative data.

This study aimed to project the future demand for lower limb arthroplasties in Colombia through to 2050, utilizing official surgical records and advanced statistical methods. These projections offer valuable insights into future healthcare needs, supporting more effective planning, the formulation of appropriate public policies, and the design of preventive and therapeutic strategies.

To achieve these objectives, it is necessary to analyze historical data on surgical interventions and apply a Poisson regression model to estimate trends and forecast the number of procedures required in the coming years. Relevant demographic variables likely to influence the incidence of these procedures influenced our analysis.

Beyond healthcare planning, this research aims to generate evidence to support researchers and healthcare professionals interested in optimizing care for the ageing population.

## 2. Materials and Methods

This study used an observational, retrospective longitudinal design, using official records to analyze arthroplasty trends in Colombia’s older adult population. Demographic data enhanced the projections, aligning them with population characteristics and historical patterns.

### 2.1. Data Sources and Population

To obtain official records of surgical procedures, it was necessary to have remote access to the Integrated Social Protection Information System (SISPRO) database with the researchers’ personal username and password. SISPRO is a tool developed by the Ministry of Health and Social Protection of Colombia, designed to consolidate and manage information related to the health and social protection of citizens. It comprises various subsystems that provide data on the healthcare sector, including the supply and demand of services, service quality, insurance coverage, financing, and social promotion [21]. Its subsystems include: the RUAF (Unique Registry of Affiliates), which consolidates health system affiliations; the RIPS (Individual Health Service Provision Records), which document healthcare services provided; the REPS (Special Registry of Healthcare Service Providers), where providers register their capabilities and services authorized for the Colombian health system; and the RETHUS (Health Human Talent Registry), which lists personnel authorized to practice a profession or occupation in the health sector [21,22].

In the RIPS subsystem, the researchers extracted the data using Microsoft Excel and its pivot table function, managing the following fields: “Unique Health Procedure Code (CUPS) classification”, “procedures and surgical interventions”, “musculoskeletal system”, “repair procedures and grafts in joint structures”, and “lower limb arthroplasties”. From this category, the following CUPS codes were selected: 8151—hip arthroplasty, including total hip reconstruction and arthroplasty of both femoral head and acetabulum with a prosthesis, congenital or acquired; 8152—partial hip arthroplasty, including bipolar endoprosthesis; 8153—hip arthroplasty revision, either partial or total; 8154—knee arthroplasty; 8155—knee arthroplasty revision; 8156—total ankle prosthetic arthroplasty; 8157—foot and toe arthroplasty; and 8158—ankle arthroplasty revision.

For analytical purposes, the report separated primary arthroplasties—specifically knee (code 8154) and hip arthroplasties (codes 8151 and 8152). Revision procedures (codes 8153, 8155, 8158) and other types of joint replacements (codes 8156 and 8157) fall under the category “other arthroplasties/revisions”. This classification enabled a distinction between primary joint replacements and revision surgeries in the analysis.

The “patients attended” filter was indispensable to avoid data duplication in cases where an individual received treatment more than once for the same condition or underwent the same procedure multiple times. Age group data were stratified into five-year intervals starting at age 60 and disaggregated by sex. The analysis did not apply any clinical exclusion criteria. However, the analysis included only complete records containing all relevant fields—year, age, sex, and procedure code. Incomplete entries were excluded. The high completeness of the data prevented the need for imputation.

To project the number of arthroplasties, population projections for individuals aged 60 and over from 2018 to 2050, generated by Colombia’s National Statistics Department (DANE), were used [23].

The authors extracted the data directly through institutional access authorized by the Colombian Ministry of Health and Social Protection. This study did not involve primary data collection or third-party data gathering teams. SISPRO is populated with routine service data reported by healthcare providers nationwide, including both public and private institutions, as mandated by Colombian health regulations. This ensures the comprehensiveness and representativeness of the dataset.

### 2.2. Data Analysis

The projection method used a Poisson regression model based on total counts of hip and knee arthroplasties performed between 2015 and 2023 (the most recent years available in SISPRO) and applied it to population forecast datasets for individuals aged 60 and over. These datasets contain demographic growth estimates by age and sex from 2015 to 2050, according to DANE projections. The formula for the Poisson regression model was:

log(λ) = β0 + β1 ⋅ Year + β2 ⋅ Sex + β3 ⋅ Population

The variable “Population” refers to the absolute number of individuals aged 60 and over per year, without standardization per unit. Sex was mainly a covariate rather than constructing separate models for each sex. Additional models were necessary for knee, hip, and other types of arthroplasties.

We evaluated model fit through a post hoc overdispersion check using the Pearson chi-square statistic. Additionally, a limited sensitivity analysis was performed by refitting the Poisson model with data from 2018 to 2023, confirming the consistency of the main predictors. The projections assume that the relationships between year, sex, population size, and arthroplasty rates observed from 2015 to 2023 will remain stable throughout the projection period. The model also relies on official population forecasts from DANE and assumes no major disruptions in clinical practice, health policy, or access to surgical procedures that could substantially alter future arthroplasty trends.

All statistical analyses used R software (version 4.3.1; R Development Core Team, 2023), employing the stats, MASS, and ggplot2 packages. The code used for projections is available as Supplementary Material to ensure transparency and reproducibility.

### 2.3. Ethical Considerations

Regarding ethical requirements, this research adhered to the Declaration of Helsinki and did not involve experiments on humans or animals, nor the use of personal patient data, as it relied exclusively on anonymized, publicly available data [24]. The analysis used aggregate records from national administrative databases, which are fully de-identified and do not contain any information that allows for individual re-identification. According to Resolution 8430 of 1993 issued by the Colombian Ministry of Health and Social Protection, this type of study is considered risk-free and does not require ethical approval.

## 3. Results

### 3.1. Procedures Performed from 2015 to 2023

During the period from 2015 to 2023, a total of 8,119,296 elderly individuals received care for procedures and surgical interventions (including dental procedures); of these, 802,288 corresponded to musculoskeletal surgeries. Among these, 208,803 (26%) were fracture and dislocation reductions, and 193,603 (24.1%) involved repair procedures and grafts in joint structures. Both types of procedures showed a higher frequency among women.

Focusing on repair procedures and grafts in joint structures during the same period, lower limb arthroplasties (n = 62,728; 32.4%) accounted for a substantial share of these cases, with a higher prevalence in older women (44,766; 22.4%). The most frequently treated age groups were individuals aged 65–69 years (6.9%) and those aged ≥80 (7.4%).

**Figure 1:**
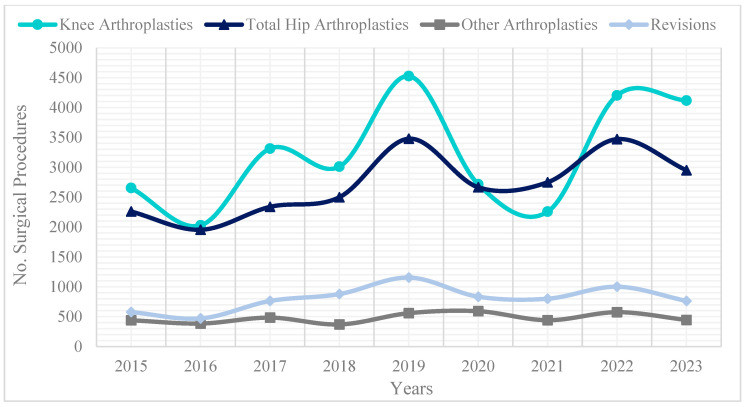
Lower Limb Arthroplasties Performed in the Elderly Population, 2015–2023, in Colombia. Note: Own elaboration based on SISPRO from Colombia data. A total of 62,728 procedures occurred in adults aged 60 and over during this period. “Other arthroplasties” include ankle, foot, or toe arthroplasties, and “Revisions” refer to repeat procedures involving the hip, knee, or ankle.

Among the 62,728 lower limb arthroplasties, 44.5% were knee arthroplasties and 37.6% hip arthroplasties. In both cases, women underwent twice as many procedures as men. Notably, a decrease was observed in 2020–2021 due to the COVID-19 pandemic (Figure 1).

Notable differences were observed between age groups and types of arthroplasties. For instance, knee arthroplasties appear the most among older adults, with the highest incidence observed in the 65–69 age group, whereas hip arthroplasties were more prominent among those aged 80 and over. This trend remained consistent across both sexes (Table 1).

### 3.2. Projection of Joint Arthroplasties, 2024–2050

The coefficients formed the basis for the projected estimates obtained from the Poisson regression models. Both year and sex were statistically significant predictors of arthroplasty counts in the general model and models stratified by procedure type. Table 2 presents the estimated coefficients, 95% confidence intervals, and *p*-values for these key variables.

A post hoc overdispersion test yielded a dispersion value of 122.16, confirming its presence. Nevertheless, the Poisson model remained in use due to the descriptive and exploratory nature of the study. This modelling choice ensured interpretability and consistency with previous projection studies using count data in orthopedic and public health research. A limited sensitivity analysis using data from 2018 to 2023 confirmed the stability of the main predictors, supporting the robustness of the projections.

**Figure 2:**
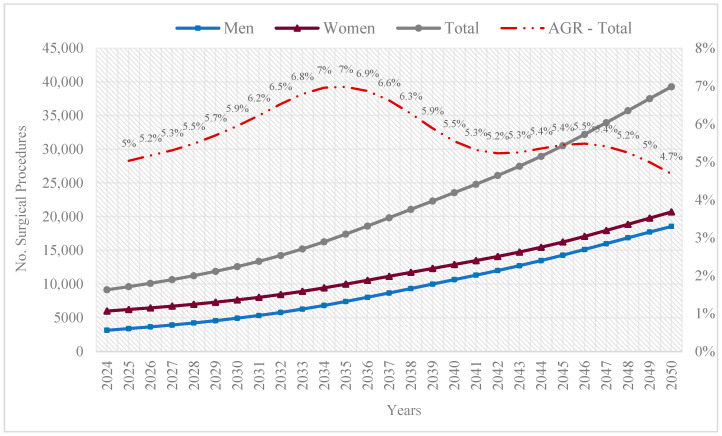
Projection and compound Annual Growth Rate of lower limb arthroplasties in the elderly population, 2024–2050, in Colombia. Note: Own elaboration based on SISPRO and DANE from Colombia data. A total of 62,728 procedures occurred in adults aged 60 and over during this period.

The overall projection indicates that by 2050, 39,270 arthroplasties are expected, reflecting a high procedural burden among individuals aged 60 and over, which 52.7% expected to be performed on women. The compound Annual Growth Rate (AGR) for the period (2024–2050) was 5.54%. Trends in projections by sex confirm a greater share among women (20,711). This is demonstrated by the regression model coefficients: β for year = 0.1545 (95% CI: 0.1348–0.1743; *p* < 0.001), indicating that each passing year increases the log count of arthroplasties. Additionally, the coefficient for sex (female vs. male) was β = 1.2735 (95% CI: 1.1848–1.3621; *p* < 0.001), indicating a higher frequency of procedures among women (Figure 2).

According to the AGR, the years with the highest growth will be 2034 and 2035, after which a decline will occur. This aspect reflects the β coefficient for the elderly population size, which slightly decreases the logarithm of the number of arthroplasties (β = −7.205 × 10^−7^; 95% CI: −8.548 × 10^−7^ to −5.863 × 10^−7^; *p* < 0.001).

**Figure 3:**
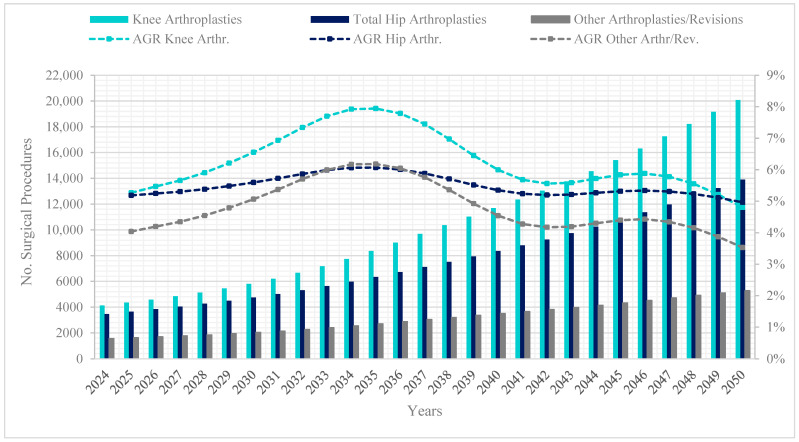
Projection and compound Annual Growth Rate by type of lower limb arthroplasty in the elderly population, 2024–2050, in Colombia. Note: Own elaboration based on SISPRO and DANE data. A total of 62,728 procedures occurred in adults aged 60 and over during this period. “Other arthroplasties/Revisions” includes ankle, foot, or toe arthroplasties, as well as revision procedures involving the hip, knee, or ankle.

When analyzing by type of arthroplasty, projections indicate that by 2050, knee arthroplasties will reach 20,084, hip arthroplasties 13,902, and other types of arthroplasties and revisions 5284. The annual growth rates highlight that hip arthroplasties will show stability in the rate after 2036, reaching slightly higher values by 2050 (5%) compared to knee arthroplasties (4.8%) (Figure 3).

This trend reflects the regression model coefficients, which indicate that each additional year increases the number of knee arthroplasties by 0.1845 (95% CI: 0.1551–0.2139; *p* < 0.001), and hip arthroplasties by 0.1745 (95% CI: 0.1451–0.2039; *p* < 0.001). In both cases, the coefficients for sex suggest that being female is associated with a higher number of arthroplasties. Meanwhile, the population coefficients indicate a decrease in the logarithm of the number of both knee arthroplasties (β =−7.205 × 10^−7^; 95% CI: −9.26 × 10^−7^ to −5.15 × 10^−7^; *p* < 0.001) and hip arthroplasties (β =−6.205 × 10^−7^; 95% CI: −8.20 × 10^−7^ to −4.21 × 10^−7^; *p* < 0.001).

## 4. Discussion

The findings of this study offer an analysis of trends in surgical procedures among Colombia’s older population and the burden of disease due to musculoskeletal conditions. The results on the proportion of procedures related to joint repairs, particularly lower limb arthroplasties, indicate a significant burden of osteoarthritis, especially among older women [25]. This aligns with global estimates predicting a notable increase in knee and hip osteoarthritis among women [26].

Similarly, the observed differences between age groups and types of arthroplasties underscore the importance of age-specific strategies. The predominance of knee arthroplasties in the 65–69 age group and hip arthroplasties in those over 80 corroborates previously reported demand patterns. These findings reflect the growth of the ageing population, particularly among nonagenarians, and underscore the need for tailored health treatments and interventions [13,27,28].

On the other hand, the impact of the COVID-19 pandemic on procedures performed during 2020–2021 revealed a marked decline, mirroring global reductions in surgical activity during the pandemic. This affected public health statistics related to osteoarticular disease treatment and the healthcare system’s capacity to meet such demand [29,30,31,32]. Particularly, the availability and accessibility of procedures such as hip and knee arthroplasties serve as indicators of how the healthcare system is working to improve the quality of life of its citizens [33]. Moreover, the pandemic highlighted the vulnerability of surgical services to external shocks, underscoring the need for resilient healthcare systems capable of maintaining access to essential procedures for the elderly population even during public health crises.

Projections to 2050 indicate an increase in the demand for arthroplasties, highlighting the need for healthcare resource planning, surgical availability, and capacity to manage the growth of the elderly population and their requirements for arthroplasties (particularly among women). The positive year coefficient (β = 0.1545) evidences this trend, reflecting the progressive increase in arthroplasties over time due to population ageing and the growing need for medical interventions. Previous studies have also highlighted this aspect, showing a worldwide increase in the demand for arthroplasties [13,14,15,34]. Furthermore, the findings underscore the importance of involving patients and the public in shaping preventive strategies and co-producing policies to promote equitable and sustainable healthcare delivery in the context of accelerated ageing.

The suitability for analyzing count data guided the choice of a Poisson regression model in this study, such as the annual number of lower limb arthroplasties, where the dependent variable represents discrete non-negative integers. Unlike linear regression, which assumes a normal distribution, Poisson regression directly models count data and ensures that predictions remain non-negative, making it particularly useful for forecasting trends in healthcare demand [35,36,37]. Epidemiological and health services research widely employs this approach and is common in several comparative studies for projecting trends in medical procedures, including hip and knee arthroplasties. For instance, research conducted in the United Kingdom, Scotland, and the United States has demonstrated the effectiveness of Poisson regression in capturing temporal and demographic variations in surgical demand [13,14,16,17,18,34].

Building on these projections, the negative population coefficient (β = −7.205 × 10^−7^) indicated, on the one hand, the demographic changes expected due to the absence of generational arthroplasty, stemming from declining fertility rates and a gradual deceleration in the growth of the older population [1,38]; and, on the other hand, a saturation in arthroplasty demand as life expectancy continues to rise [16].

Specifically, the projected decline in the compound Annual Growth Rate (AGR) of arthroplasties after 2035 may signal a saturation point, whereby the population requiring surgery stabilizes as high-risk cohorts have already undergone procedures. In parallel, the emergence of alternative non-surgical management strategies for osteoarthritis—including biological therapies, regenerative medicine, and minimally invasive joint preservation techniques—may further contribute to moderating future demand [39]. Advances in early diagnosis and conservative treatment could delay the need for surgery [40].

Moreover, demographic shifts, particularly the slowing growth of the older population, result from historically lower fertility rates, which reinforce this trend towards stabilization. These broader demographic and epidemiological dynamics also provide a useful framework for interpreting variations in demand according to arthroplasty type [13,14].

The projections obtained through the Poisson regression model directly support these interpretations, which revealed a declining growth trend after 2035. Furthermore, demographic studies have consistently shown that historically lower fertility rates contribute to a reduced growth in the elderly population, influencing healthcare demand dynamics [1,38]. Previous projection studies have also discussed the concept of saturation in healthcare demand, particularly in elective surgical procedures such as arthroplasties [17,19,20].

Regarding projections by type of arthroplasty, they reveal that the deceleration in growth rates for knee arthroplasties (more common in men) may be due to the shorter life expectancy observed in men [41]. In contrast, the greater longevity of women explains the stabilization of the annual growth rate of hip arthroplasties (more common in women) in the final projected years, eventually surpassing knee arthroplasties [16,41].

Despite the strengths and elements that contribute to recognizing surgical procedure trends in the elderly population, the Poisson regression model used has limitations that must be highlighted. Firstly, the model assumes that the variance equals the mean (λ) [42]. In practice, count data, such as arthroplasties, often exhibit overdispersion, which can lead to underestimations of uncertainty and unreliable coefficients. A post hoc overdispersion test using the Pearson chi-square statistic assessed this issue, yielding a dispersion value of 122.16. Despite the presence of overdispersion, the descriptive and exploratory nature of the study justified maintaining the Poisson model. Additionally, by including only the population aged 60 and over as an explanatory variable, other important factors are overlooked, such as socioeconomic conditions, advancements in surgical techniques, and policy changes that could influence the number of arthroplasties. Consequently, the model may not fit adequately [42]. Finally, with only nine years of available data, the sample may not fully reflect long-term trends [43].

One additional limitation of this study is that the available data did not allow adjustment for individual clinical variables, such as comorbidities or hospital type. Consequently, the projections are based exclusively on demographic characteristics (age, sex) and procedural counts, which may limit the model’s sensitivity to clinical variability. Moreover, recent studies suggest that environmental factors such as climatic conditions may also influence musculoskeletal health outcomes and care demands, particularly in ageing populations [44].

A further limitation concerns underreporting, miscoding, and variations in reporting practices over time, which can affect the use of administrative data. Although SISPRO is a national-level source that integrates multiple health subsystems, its quality depends on institutional reporting, which may introduce uncertainty in procedure counts.

Nevertheless, the findings of this study provide an approximation of the demand for arthroplasties among the elderly population and contribute important insights for public health policy. For instance, the increase in the elderly population highlights the importance of developing care, rehabilitation, and preventive programs that can reduce the need for arthroplasties through early management of musculoskeletal diseases, and measures for cost planning and service distribution over time [45]

Furthermore, fostering a learning curve for both practicing surgeons and those in training will be essential. This will establish a well-prepared and competent generational transition to meet the needs of an ageing population [46]. It also facilitates improved clinical outcomes, optimizes healthcare resources, enables the adoption of new technologies, assures quality of care, and ultimately enhances the quality of life for elderly patients.

Finally, although this study focused on Colombia, the methodology employed, based on national administrative health records and Poisson regression modelling, could be adapted for use in other middle-income countries with similar healthcare reporting systems. Countries with centralized procedure registries and demographic projections may replicate this approach to estimate future surgical demand, supporting cross-national comparisons and strengthening regional planning for ageing populations.

## 5. Conclusions

This study offers a detailed analysis of the current trends and future projections for lower limb arthroplasties in Colombia’s elderly population. The findings reveal a sustained increase in procedure volumes through to 2050, with a notable predominance among women and individuals aged 65–69 and those aged 80 and over. However, projections also indicate a potential deceleration in growth rates after 2035, suggesting a future saturation of demand influenced by demographic shifts and the emergence of non-surgical alternatives.

These results underscore the urgent need for strategic healthcare planning to expand surgical capacity, optimize resource allocation, and implement preventive programs to delay the progression of musculoskeletal disorders. Public health policies should anticipate the evolving needs of an ageing population by strengthening orthopedic services, integrating early detection and conservative management, and promoting rehabilitation strategies to enhance quality of life.

Future research should explore the clinical, socioeconomic, and technological factors that may further influence the demand for arthroplasties, as well as the potential impact of innovations in joint preservation and regenerative therapies. Additionally, building resilient surgical and rehabilitation services will help to safeguard access to care for older adults in the face of future public health challenges.

## Figures and Tables

**Table 1:** Hip and Knee Arthroplasties Performed in the Elderly Population, 2015–2023 in Colombia.

Type of Arthroplasty	Sex	Age Group					Total > 60
		60–64	65–69	70–74	75–79	≥80	
Hip Arthroplasties	Women	2412	2852	2905	2857	5128	16,154
	Men	1729	1788	1564	1282	1846	8209
Knee Arthroplasties	Women	4261	5394	5071	3711	2269	20,706
	Men	1839	2059	1848	1436	939	8121
Total		10,241	12,093	11,388	9286	10,182	53,190

Note: Own elaboration based on SISPRO from Colombia data.

**Table 2:** Poisson Regression Coefficients for Year and Sex by Type of Arthroplasty, Colombia, 2015–2050.

Model Type	Variable	Coeff. (β)	Standard Error	95% CI	*p*-Value
General	Year	0.1545	0.0101	[0.1348, 0.1743]	<0.001
	Sex (Women = 1)	1.2735	0.0452	[1.1848, 1.3621]	<0.001
Rodilla	Year	0.1845	0.015	[0.1551, 0.2139]	<0.001
	Sex (Women = 1)	1.2735	0.065	[1.1461, 1.4009]	<0.001
Hip Arthroplasties	Year	0.1745	0.015	[0.1450, 0.2039]	<0.001
	Sex (Women = 1)	1.1735	0.07	[1.0363, 1.3107]	<0.001
Other Arthroplasties	Year	0.1945	0.02	[0.1553, 0.2337]	<0.001
	Sex (Women = 1)	1.3735	0.09	[1.1970, 1.5499]	<0.001

Note: All models were estimated using official arthroplasty records and population projections. Sex was coded as 1 for women and 0 for men. All *p*-values < 0.001.

## Data Availability

Part of the data supporting the reported results are available through publicly archived datasets provided by the National Administrative Department of Statistics (DANE), including the National Population Projections and Retroprojections for 1950–2019 and 2020–2070, based on the 2018 National Population and Housing Census (available at https://www.dane.gov.co/index.php/estadisticas-por-tema/demografia-y-poblacion/proyecciones-de-poblacion, accessed 20 June 2024). Additionally, remote access to official surgical procedure records was granted via the Integrated Social Protection Information System (SISPRO) with the researcher’s credentials.

## Supplementary Materials

The following supporting information can be downloaded at: https://www.mdpi.com/article/10.3390/epidemiologia6020024/s1, File S1: [Supplementary File 1_poisson_regression_script] used for Poisson regression modelling and projections of lower limb arthroplasties (2015–2050).

## Abbreviations

AGR	Compound Annual Growth Rate
CUPS	Unique Health Procedure Code
DALYs	Disability-Adjusted Life Years
DANE	National Administrative Department of Statistics
GBD	Global Burden of Disease, Injuries, and Risk Factors
RETHUS	Health Human Talent Registry
REPS	Special Registry of Healthcare Service Providers
RUAF	Unique Registry of Affiliates
RIPS	Individual Health Service Provision Records
SISPRO	Integrated Social Protection Information System
YLDs	Years Lived with Disability

## References

[1] Magnus G. (2013). La era del Envejecimiento: Cómo la Demografía Está Transformando la Economía Global y Nuestro Mundo. Oceano.

[2] World Health Organization (2022). Ageing and Health. https://www.who.int/news-room/fact-sheets/detail/ageing-and-health.

[3] World Health Organization (2022). Musculoskeletal Health. https://www.who.int/news-room/fact-sheets/detail/musculoskeletal-conditions.

[4] James S.L.G., Abate D., Abate K.H., Abay S.M., Abbafati C., Abbasi N., Abbastabar H., Abd-Allah F., Abdela J., Abdelalim A. (2018). Global, regional, and national incidence, prevalence, and years lived with disability for 354 Diseases and Injuries for 195 countries and territories, 1990–2017: A systematic analysis for the Global Burden of Disease Study 2017. Lancet.

[5] Liu S., Wang B., Fan S., Wang Y., Zhan Y., Ye D. (2022). Global burden of musculoskeletal disorders and attributable factors in 204 countries and territories: A secondary analysis of the Global Burden of Disease 2019 study. BMJ Open.

[6] Steinmetz J.D., Culbreth G.T., Haile L.M., Rafferty Q., Lo J., Fukutaki K.G., Singh S. (2023). Global, regional, and national burden of osteoarthritis, 1990–2020 and projections to 2050: A systematic analysis for the Global Burden of Disease Study 2021. Lancet Rheumatol.

[7] Atukorala I., Hunter D.J. (2023). A review of quality-of-life in elderly osteoarthritis. Expert Rev. Pharmacoecon. Outcomes Res..

[8] Taljanovic M.S., Jones M.D., Hunter T.B., Benjamin J.B., Ruth J.T., Brown A.W., Sheppard J.E. (2003). Joint Arthroplasties and Prostheses. RadioGraphics.

[9] Kelmer G., Stone A.H., Turcotte J., King P.J. (2021). Reasons for Revision: Primary Total Hip Arthroplasty Mechanisms of Failure. J. Am. Acad. Orthop. Surg..

[10] Lei P.F., Hu R.Y., Hu Y.H. (2019). Bone Defects in Revision Total Knee Arthroplasty and Management. Orthop. Surg..

[11] Siviero P., Marseglia A., Biz C., Rovini A., Ruggieri P., Nardacchione R., Maggi S. (2020). Quality of life outcomes in patients undergoing knee replacement surgery: Longitudinal findings from the QPro-Gin study. BMC Musculoskelet. Disord..

[12] Evans J.T., Evans J.P., Walker R.W., Blom A.W., Whitehouse M.R., Sayers A. (2019). How long does a hip replacement last? A systematic review and meta-analysis of case series and national registry reports with more than 15 years of follow-up. Lancet.

[13] Matharu G.S., Culliford D.J., Blom A.W., Judge A. (2022). Projections for primary hip and knee replacement surgery up to the year 2060: An analysis based on data from The National Joint Registry for England, Wales, Northern Ireland and the Isle of Man. Ann. R Coll. Surg. Engl..

[14] Culliford D., Maskell J., Judge A., Cooper C., Prieto-Alhambra D., Arden N.K. (2015). Future projections of total hip and knee arthroplasty in the UK: Results from the UK Clinical Practice Research Datalink. Osteoarthr. Cartil..

[15] Lee K., Goodman S.B. (2008). Current state and future of joint replacements in the hip and knee. Expert Rev. Med. Devices.

[16] Shichman I., Roof M., Askew N., Nherera L., Rozell J.C., Seyler T.M., Schwarzkopf R. (2023). Projections and Epidemiology of Primary Hip and Knee Arthroplasty in Medicare Patients to 2040–2060. JBJS Open Access..

[17] Sloan M., Premkumar A., Sheth N.P. (2018). Projected volume of primary total joint arthroplasty in the U.S., 2014 to 2030. J. Bone Jt. Surg.–Am..

[18] Wagner E.R., Farley K.X., Higgins I., Wilson J.M., Daly C.A., Gottschalk M.B. (2020). The incidence of shoulder arthroplasty: Rise and future projections compared with hip and knee arthroplasty. J. Shoulder Elb. Surg..

[19] Pabinger C., Lothaller H., Portner N., Geissler A. (2018). Projections of hip arthroplasty in OECD countries up to 2050. HIP Int..

[20] Patel A., Pavlou G., Mújica-Mota R.E., Toms A.D. (2015). The epidemiology of revision total knee and hip arthroplasty in England and Wales: A comparative analysis with projections for the United States. a study using the national joint registry dataset. Bone Jt. J..

[21] Ministerio de Salud y Protección Social (2024). SISPRO-Sistema Integrado de Información de la Protección Social. https://www.sispro.gov.co/Pages/Home.aspx.

[22] Rosselli D., Pantoja-Ruiz C., Rosselli D., Pantoja-Ruiz C. (2022). SISPRO: La base de datos administrativa del sistema de salud colombiano. Acta Neurológica Colomb..

[23] Departamento Nacional de Estadística de Colombia (DANE) (2023). Proyecciones de Población. https://www.dane.gov.co/index.php/estadisticas-por-tema/demografia-y-poblacion/proyecciones-de-poblacion.

[24] World Medical Association (2024). World Medical Association Declaration of Helsinki: Ethical Principles for Medical Research Involving Human Participants. JAMA.

[25] Hawker G.A. (2019). Osteoarthritis is a serious disease. Clin. Exp. Rheumatol..

[26] Cross M., Smith E., Hoy D., Nolte S., Ackerman I., Fransen M., Bridgett L., Williams S., Guillemin F., Hill C.L. (2014). The global burden of hip and knee osteoarthritis: Estimates from the Global Burden of Disease 2010 study. Ann. Rheum. Dis..

[27] Rubin L.E., Blood T.D., Defillo-Draiby J.C. (2016). Total Hip and Knee Arthroplasty in Patients Older Than Age 80 Years. J. Am. Acad. Orthop. Surg..

[28] Jauregui J.J., Boylan M.R., Kapadia B.H., Naziri Q., Maheshwari A.V., Mont M.A. (2015). Total Joint Arthroplasty in Nonagenarians: What Are the Risks?. J. Arthroplast..

[29] Mylonakis A., Kalfoutzou A., Panagakis A., Despotidis M., Yfantopoulos J., Mylonakis A., Kalfoutzou A., Mylonakis A., Despotidis M., Yfantopoulos J. (2022). The Impact of the COVID-19 Pandemic on Surgical Activities: A Single-Center Experience and Literature Review. Cureus.

[30] Okabaiasse Luizeti B., Augusto Santos Perli V., Gonçalves da Costa G., da Conceição Eckert I., Marino Roma A., Miura da Costa K. (2021). Impact of the COVID-19 pandemic on surgical procedures in Brazil: A descriptive study. medRxiv.

[31] Hübner M., Zingg T., Martin D., Eckert P., Demartines N. (2020). Surgery for non-Covid-19 patients during the pandemic. PLoS ONE.

[32] Dobbs T.D., Gibson J.A.G., Fowler A.J., Abbott T.E., Shahid T., Torabi F., Griffiths R., Lyons R.A., Pearse R.M., Whitaker I.S. (2021). Surgical activity in England and Wales during the COVID-19 pandemic: A nationwide observational cohort study. Br. J. Anaesth..

[33] Konopka J.F., Lee Y.Y., Su E.P., McLawhorn A.S. (2018). Quality-Adjusted Life Years After Hip and Knee Arthroplasty Health-Related Quality of Life After 12,782 Joint Replacements. JBJS Open Access.

[34] Farrow L., McLoughlin J., Gaba S., Ashcroft G.P. (2022). Future demand for primary hip and knee arthroplasty in Scotland. Musculoskelet. Care.

[35] Lawless J.F. (1987). Regression methods for poisson process data. J. Am. Stat. Assoc..

[36] Cleophas T.J., Zwinderman A.H. (2018). More on Poisson Regressions. Regression Analysis in Medical Research.

[37] Coxe S., West S.G., Aiken L.S. (2009). The Analysis of Count Data: A Gentle Introduction to Poisson Regression and Its Alternatives. J. Pers. Assess..

[38] United Nations Department of Economic and Social Affairs PD (2024). World Population Ageing 2023: Challenges and Opportunities of Population Ageing in the Least Developed Countries. https://desapublications.un.org/publications/world-population-ageing-2023-challenges-and-opportunities-population-ageing-least.

[39] Gobbi A., Herman K., Szwedowski D., Gobbi A., Herman K., Szwedowski D. (2023). Bio-Orthopedics: A New Approach to Osteoarthritis and Joint Disorders. Cartilage Disorders-Recent Findings and Treatment.

[40] Hendriks J. (2019). Saving the Joint: New Methods for Early Diagnosis and Treatment. Ph.D. Thesis.

[41] Fernández-Ortiz Y.N. (2024). Healthy Aging in Colombia 2018 and Its Variation in Relation to Social Conditions. Int. J. Environ. Res. Public Health.

[42] Kamalja K.K., Wagh Y.S. (2022). Estimation in Zero-Inflated Generalized Poisson Distribution. J. Data Sci..

[43] Loehle C., Arghami N. (2017). Bounded random walks as a null model for evaluating population trends. Popul. Ecol..

[44] Cuenca-Zaldívar J.N., del Corral-Villar C., García-Torres S., Araujo-Zamora R., Gragera-Peña P., Martínez-Lozano P., Sánchez-Romero E.A. (2025). Fourteen-Year Retrospective Cohort Study on the Impact of Climatic Factors on Chronic Musculoskeletal Pain: A Spanish Primary Care Analysis. Int. J. Rheum. Dis..

[45] Fernández Ortiz Y.N., Peñaloza Quintero R.E. (2025). Social health inequalities in healthy ageing, Colombia 2018. Discov. Soc. Sci. Health.

[46] Rosengart T.K., Doherty G., Higgins R., Kibbe M.R., Mosenthal A.C. (2019). Transition Planning for the Senior Surgeon: Guidance and Recommendations from the Society of Surgical Chairs. JAMA Surg..

